# Mr. Burns, on the Structure of Parts in Crural Hernia

**Published:** 1807-01

**Authors:** Allen Burns

**Affiliations:** Glasgow


### To the Editors of the Medical and Physical Journal.

GENTLEMEN,

IN July last, I published in the Edinburgh Medical and Surgical Journal, an account of the structure of the parts concerned in the operation for crural hernia. In your Journal for August, which I did not meet with till now, I find an anonymous criticism on my paper. In this I am accused of plagiarism from Mr. Astley Cooper, and from Mr. Hey, and am censured for not acknowledging my obligations to these gentlemen.

A short statement of facts will prove how far I have acted wrong. In the summer of 1802, my brother, who was then a teacher of anatomy, and myself were engaged in dissecting the parts concerned in the operation for femoral hernia. While examining the structure of these parts in an emaciated and anasarcous female, we ascertained the mode of junction of the fascia of the thigh with Poupart's ligament to be such as I formerly described. At the time my brother made out an account of the principal facts, which he transmitted in August, 1802, to that ingenious and learned anatomist, Mr. Abernethy, whose letter in return is dated August 31, 1802. From this description I selected most of the particulars with respect to the structure of the parts, but the pathological inferences I added at a latter period. I made before the winter of 1802 several other dissections of a similar kind, and had procured a variety of preparations illustrative of what we conceived to be discoveries of our own. These my brother publickly demonstrated to his class in December, 1802.

This statement will, I imagine, be a sufficient answer to the charge of plagiarism from either Mr. Hey or Mr. Cooper, for both of whom, on account of their eminent abilities and successful labors for the advancement of surgical knowledge, I entertain the highest respect. It may not here be improper to mention, that the femoral ligament of Mr. Hey is essentially different from the falciform process of the fascia vera. Mr. Hey, in describing the femoral ligament, states that, "as the finger passes downwards through the crural foramen," it will be pressed upon a part of the fascia, more compact than the rest, which runs transversely about three-eighths of an inch below Poupart's ligament, and sometimes presents a sharp edge

 to to the finger. It is not situated in the same plane with the ligament of Poupart but lies deeper, that is, at a greater distance from the integuments." Practical Observations on Surgery, p. 152 et seq. The falciform process of the fascia I describe as follows, "We find that the firm or internal layer of the fascia, the fascia vera, stops just at the entrance of the vena saphena, and in many cases it terminates abruptly with a neat firm margin, which is traced some way down the thigh. The edge is lunated and the concavity is directed toward the pubes or superficial vein."

In so far as I can judge, the femoral ligament described by Mr. Hey is just that slip of the pectineal aponeurosis, which rises up between the vena saphena and the deep crural vein to join the falciform process of the fascia. It is felt, as Mr. Hey states, extending across the thigh when the finger is forced through the crural aperture, and it "lies deeper" than the ligament of Poupart, "that is, at a greater distance from the integuments." To discover the falciform process of the fascia, the finger is to be pushed from the abdomen through the foramen crurale. Having accomplished this, instead of carrying it down, so as to come in contact with the femoral ligament of Mr. Hey, its point is to be curved upward and outward toward the spine of the ischii, when the crescentia falciform process of the fascia will be more or less distinctly felt interrupting its farther progress.

Mr. Hey published his excellent Observations on Surgery in 1803, and Mr. Astley Cooper favoured the world with his ingenious and splendid work on inguinal hernia in 1804. Yet the author of the observations has the ingenuity to discover, that I am under peculiar obligations for my description to both of these gentlemen. I extracted, as he asserts, from their works, the substance of what was written fully a year before either of them was published. I am forsooth guilty of plagiarism from books which I had not then seen, and which the public had not perused; and what is still more astonishing, the learned author has traced a complete coincidence in sentiment and expression in description, which to every other person must appear as distinct from each other as it is possible for any two things to be. Let any one compare Mr. Hey's description of the femoral ligament with the account which I have published of the falciform process of the facia lata; and if he can discover a greater similarity in the detail than what is occasioned by both employing English words to to express their ideas, I give him credit for his penetration. Mr. Hey describes a process which runs transverse; I, one running, according to the length of the thigh; yet the love of misrepresentation, or ignorance of the subject, has so far misled the author as to make him assert that we are both describing one and the same process, which cannot surely be admitted, unless it be granted, that transverse and longitudinal have become synonimous terms.

It only remains for me to apologise to Mr. Abernethy, who, on this occasion will, I trust, excuse the freedom I have taken, in twice introducing his name. For the frequent repetition of the names of Mr. Cooper and Mr. Hey, I also feel it necessary to request their indulgence. To both gentlemen I may add, that although I do not consider myself as indebted to their valuable works for any of the information contained in my pages, that yet, as a sense of justice to their great talents, I would, had I at the time that I sent my observations to Edinburgh, had an opportunity of consulting their publications, have mentioned, in due terms, any points wherein they and I coincided in description.

I am, &c.

*Glasgow, Oct.* 31, 1806. ALLEN BURNS.

